# Erythropoietin as a Neuroprotective Drug for Newborn Infants: Ten Years after the First Use

**DOI:** 10.3390/antiox11040652

**Published:** 2022-03-28

**Authors:** Serafina Perrone, Chiara Lembo, Federica Gironi, Chiara Petrolini, Tiziana Catalucci, Giulia Corbo, Giuseppe Buonocore, Eloisa Gitto, Susanna Maria Roberta Esposito

**Affiliations:** 1 Department of Medicine and Surgery, University of Parma, 43126 Parma, Italy; cpetrolini@ao.pr.it (C.P.); susannamariaroberta.esposito@unipr.it (S.M.R.E.); 2 Department of Molecular and Developmental Medicine, University of Siena, 53100 Siena, Italy; lmb.chr@gmail.com (C.L.); fedegironi@icloud.com (F.G.); t.catalucci88@gmail.com (T.C.); giulia.corbo793@gmail.com (G.C.); giuseppe.buonocore@unisi.it (G.B.); 3 Department of Human Pathology in Adult and Developmental Age “Gaetano Barresi”, University of Messina, 98125 Messina, Italy; e.gitto@unime.it

**Keywords:** erythropoietin, newborn, neuroprotection, preterm, Hypoxic Ischemic Encephalopathy, perinatal stroke

## Abstract

Protective strategies against perinatal brain injury represent a major challenge for modern neonatology. Erythropoietin (Epo) enhances endogenous mechanisms of repair and angiogenesis. In order to analyse the newest evidence on the role of Epo in prematurity, hypoxic ischemic encephalopathy (HIE) and perinatal stroke, a critical review using 2020 PRISMA statement guidelines was conducted. This review uncovered 26 clinical trials examining the use of Epo for prematurity and brain injury-related outcomes. The effects of Epo on prematurity were analysed in 16 clinical trials. Erythropoietin was provided until 32–35 weeks of corrected postnatal age with a dosage between 500–3000 UI/kg/dose. Eight trials reported the Epo effects on HIE term newborn infants: Erythropoietin was administered in the first weeks of life, at different multiple doses between 250–2500 UI/kg/dose, as either an adjuvant therapy with hypothermia or a substitute for hypothermia. Two trials investigated Epo effects in perinatal stroke. Erythropoietin was administered at a dose of 1000 IU/kg for three days. No beneficial effect in improving morbidity was observed after Epo administration in perinatal stroke. A positive effect on neurodevelopmental outcome seems to occur when Epo is used as an adjuvant therapy with hypothermia in the HIE newborns. Administration of Epo in preterm infants still presents inconsistencies with regard to neurodevelopmental outcome. Clinical trials show significant differences mainly in target population and intervention scheme. The identification of specific markers and their temporal expression at different time of recovery after hypoxia-ischemia in neonates might be implemented to optimize the therapeutic scheme after hypoxic-ischemic injury in the developing brain. Additional studies on tailored regimes, accounting for the risk stratification of brain damage in newborns, are required.

## 1. Introduction—Description of the Condition

*What is Known?*

- Neonates with moderate or severe hypoxic-ischemic encephalopathy treated with therapeutic hypothermia still experience devastating complications.
- Improvement in the quality of preterm neonatal care has drastically reduced mortality, but morbidity is still high in preterm babies with developmental delay, cerebral palsy, hearing and vision problems.
- Strokes which occur early during the brain development still represent a challenge in terms of learning causality and optimizing the outcomes
- Preclinical studies with optimal animal models and pharmacokinetic- pharmacodynamic modelling have demonstrated neuroprotective effects of erythropoietin

*What is New?*

- Erythropoietin shows promise in conjunction with therapeutic hypothermia, in neonatal hypoxic-ischemic encephalopathy
- Studies on very preterm newborns treated with Epo highlighted an improved white matter development
- There is a need for optimal dose-ranging studies and tailored regimes, accounting for patients’ risk of brain damage

### 1.1. Neurological Impairment in Neonatal Population

Hypoxic ischemic encephalopathy (HIE) is the most common cause of neonatal brain injury in developed countries, followed by the damage linked to prematurity and neonatal strokes [1]. In the last decades, developments in neonatal care have led to a substantial increase in survival, which at the same time has led to an increase of a broad spectrum of non-fatal neurological complications [1]. In this context, recent research has focused on the development of neuroprotective strategies in order to limit brain damage and improve the quality of life [2]. Erythropoietin (Epo) has been proven to be a potential neuroprotective agent [2]. It has been mostly studied in preterm infants as it was primarily administered as a treatment for anaemia of prematurity, but new trials also have investigated its role in HIE and neonatal stroke [2].

### 1.2. Pathogenesis of Brain Damage 

#### 1.2.1. Preterm Infants and Brain Damage

According to the World Health Organization, prematurity is defined as birth before 37 completed weeks of gestation [3]. The prevalence of preterm birth is estimated at 11.1% globally, decreasing to 5% in some European countries and reaching 18% in certain African countries [4]. Low income-countries present higher rates of prematurity and account for the 60% of the world’s preterm births [4]. However, some higher income countries have shown an increase in preterm birth rates in the last years, as a result of the lower threshold of fetal viability and a reduction in stillbirth rate [4,5,6].

Despite the increase in survival, the majority of premature infants still present an increased morbidity with a wide array of complications both in the short and long-term [7,8]. The major short term complications of prematurity are represented by respiratory diseases, such as bronchopulmonary dysplasia and respiratory distress syndrome, necrotizing enterocolitis, feeding problems, sepsis and neurosensory defects [9]. Short term neurological complications include periventricular leukomalacia, seizures, intraventricular haemorrhage, cerebral palsy and HIE [9,10]. In the long term, premature babies have shown an increased rate of hospitalization and different grades of neurodevelopmental impairment. Severe deficits include cerebral palsy, significant visual and hearing loss and poor intellectual quotient, usually detected within the first two years of age. Mild to moderate deficits refer to behavioural problems, learning impairments and social integration, more often highlighted during school-age [7,11,12,13]. As far as neuropathology is concerned, the complexity of brain abnormalities in premature infants is described as encephalopathy of prematurity [14]. The main feature of encephalopathy of prematurity is characterized by diffuse white matter injury, which is strongly related to neurodevelopmental impairment [15]. In particular, findings in preterm infants brains have shown diffuse microscopic punctate lesions, a decrease in white matter volume and thinner white matter tracts [16,17]. These alterations are caused by defects in myelination, which is essential for brain connectivity as it grants a rapid transmission of the action potential and protects axons [18]. The myelination process starts at 34 weeks of gestation and proceeds during the first years of life until 30 years of age [19,20,21]. The main role in the myelination process is played by oligodendrocytes, specific type of neuroglia cells which migrate in specific areas of the developing brain during the third trimester of pregnancy [22]. Thus, myelinization appears to be particularly vulnerable in the last months of pregnancy [23,24]. Consequently, perinatal damage and premature birth interfere with oligodendrocytes physiological development leading to preterm brain injury [23,24]. Furthermore, brain damage in premature babies is also represented by grey matter injury, in particular in the cerebral and cerebellar cortex, thalamus, hippocampus and basal ganglia [25]. In the last few it represents the most frequent years, increasing evidence has highlighted the cerebellar involvement in encephalopathy of prematurity [26]. The cerebellum presents a rapid increase in volume and complexity during the last weeks of gestation, hence a premature arrest of its development may lead to adverse neurodevelopmental outcomes [27,28].

#### 1.2.2. Hypoxic Ischemic Encephalopathy

A significant part of the investigations about neuroprotective effects of Epo has focused on HIE, as it represents the most frequent cause of brain injury in term newborns [1]. The incidence is estimated approximately 2 per 1000 live births in developed countries and 26 per 1000 live births in low-resource countries [29]. In this context, the discovery of therapeutic hypothermia (TH) has determined an essential change in the prognosis, contributing to a huge decrease of morbidity after the ischemic event [30,31]. Nevertheless, this approach is reserved to term neonates and has only a partial effectiveness, because 45% of patients still die or have neurodevelopmental disability despite the treatment [32]. In addition, the use of TH remains significantly lower in developing countries compared to high-resource countries [33]. Therefore, new adjuvant therapies have been proposed with the aim to determine neuroprotective effects alone or combined with TH [34]. A better understanding of HIE pathophysiology has helped to introduce different types and timings of treatments [35]. In the pathogenesis of HIE injury, four phases can be recognized [35]. The primary phase is characterized by the acute hypoxic ischemia which causes a primary energy failure, with consequent decrease of ATP, increase of lactate levels and systemic acidosis [36,37]. This process generates a dysfunction of the Na/K ATPase, with a consequent increase of intracellular Na which leads to intracellular oedema and cell lysis [36,37]. Moreover, under a condition of lack of substrates for respiration and oxidative phosphorylation, the ATPase can act in the opposite direction by consuming ATP to maintain the transmembrane mitochondrial potential, causing additional acidification of the cytoplasm [38,39]. 

This mechanism initiates the cascade of excitotoxic damage [38,39]. The subsequent latent phase lasts from 1 to 6–24 h and is characterized by a transitory increase in oxygenation, with a partial recovery of the energy metabolism [40,41]. This period offers an initial therapeutic window to limit further neuronal damage [34]. In fact, therapeutic hypothermia is recommended in this time frame, typically before 6 h from birth [42]. Subsequently, a secondary energy failure occurs. In this phase the damage is perpetrated by the mechanisms of excitotoxicity, oxidative stress, mitochondrial damage, inflammation and cell death by necrosis and apoptosis [37]. Finally, the tertiary phase lasts for months to years after the hypoxic insult [43]. This is characterized by a persistent damage leading to reactive gliosis, persistent inflammation and epigenetic changes, coexisting with a component of reparation and regeneration [43].

#### 1.2.3. Perinatal and Neonatal Stroke

Neonatal stroke is defined as a cerebrovascular event occurring in the first 28 days of life, while perinatal stroke also includes the fetal period after 20 weeks of gestation [44]. Perinatal stroke has an estimated incidence of 1:1600–1:2300 live births [45]. The stroke can be both an arterial or venous process, with a haemorrhagic, ischemic or mixed component [45,46]. Acute symptomatic perinatal strokes account for the 50% of perinatal strokes [47]. The neonatal arterial ischemic stroke is the most frequent, followed by cerebral sinovenous thrombosis and neonatal haemorrhagic stroke [47]. The most common clinical presentation in the first weeks of life is represented by seizures [48,49]. Some perinatal strokes, such as an arterial presumed perinatal ischemic stroke, periventricular venous infarction and presumed neonatal haemorrhagic stroke, have a delayed presentation after the 28th day of life [47]. Long term morbidities of perinatal strokes are represented by epilepsy, cerebral palsy, congenital hemiplegia and neurodevelopmental impairment, including intellectual disability, language retardation and behavioural problems from infancy to later life [50,51,52]. Studies on animal models have described two phases of the damage caused by perinatal stroke: the first one is represented by the acute injury which determines a core of rapid cell-death; the second phase is characterized by the perpetration of the damage in the peri-infarct region during the following days or weeks [36]. The peri-infarct region is particularly vulnerable and can be potentially preserved by the activation of endogenous mechanisms, still not entirely understood [53]. These may include neoangiogenesis which allows neuronal progenitor migration in the peri-ischemic area [54].

### 1.3. Erythropoietin (Epo) Actions and Neuroprotective Effects

Erythropoietin is a 30.4 kDa glycoprotein and pleiotropic cytokine, first described by Carnot in 1906 and isolated by Goldwasser and Kung in 1971, successfully produced for clinical use [55]. During fetal life Epo is produced by the liver, while after birth the production progressively takes place in the peritubular cells of the kidney [56]. Erythropoietin is primarily known for the haematological functions. After an ischemic insult lasting at least 30 min, the transcription of the Hypoxia Inducible Transcription Factor induced by hypoxia determines an increase of Epo hormone production in the kidney [57,58]. Binding of Epo to receptors on erythroid progenitor cells causes an increase in red blood cell mass. The enhanced oxygen-carrying capacity of the blood suppress the further expression of Epo completing the feedback loop [59].

The endogenous levels of Epo have been reported to increase in the brain for four hours after hypoxic exposure [60]. Hypoxia induce hypoxia-inducible factor-1, which determines the expression of growth factors such as the vascular endothelial growth factor (VEGF) and Epo [61]. The immediate effect of Epo is represented by an augmented expression of haemoglobin which improves the oxygen consumption and storage in the hypoxic tissue [59]. Therefore, high serum or amniotic fluid concentrations of Epo may suggest chronic hypoxia in newborns [62]. However, the lack of brain perfusion and oxygenation which occurs in brief periods, but still severe enough to cause brain injury, may not trigger endogenous Epo production [63]. Erythropoietin exerts intracellular protective effects after the ischemia-reperfusion damage, such as decreasing apoptosis, oxidative stress and Blood Brain Barrier (BBB) injury [64]. Furthermore, Epo has been proved to be able to stimulate angiogenesis, neurogenesis and neuronal plasticity after the ischemic damage [65,66]. 

**Figure 1:**
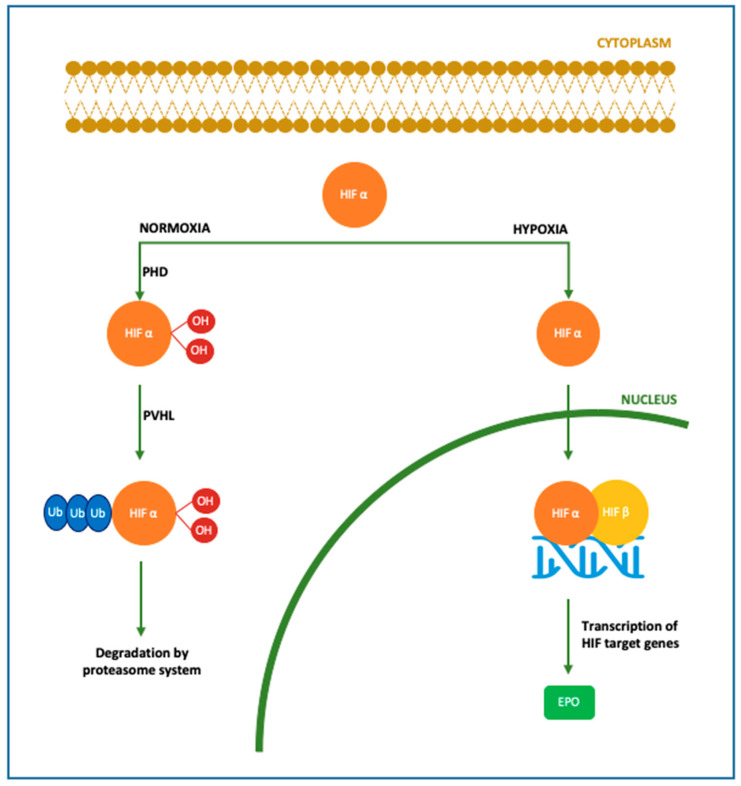
Molecular mechanism of Epo production. In the brain Epo production is upregulated by oxygen levels mainly in astrocytes, followed by oligodendrocytes, endothelial cells and microglia. In case of normoxia, cytoplasmic HIFα is hydroxylated and polyubiquitinated by PHD and pVHL, respectively. In this form, HIF is degraded by the proteasome system. In case of hypoxia, HIFα is dehydroxylated and deubiquinated, thus able to translocate into the nucleus and bind HIF β, inducing the transcription of its target genes among which Epo gene [70,71,72,97]. *HIF: hypoxia-inducible factor; PHD: prolyl-4-hydroxylases; PVHL: von Hippel–Lindau protein*.

**Figure 2:**
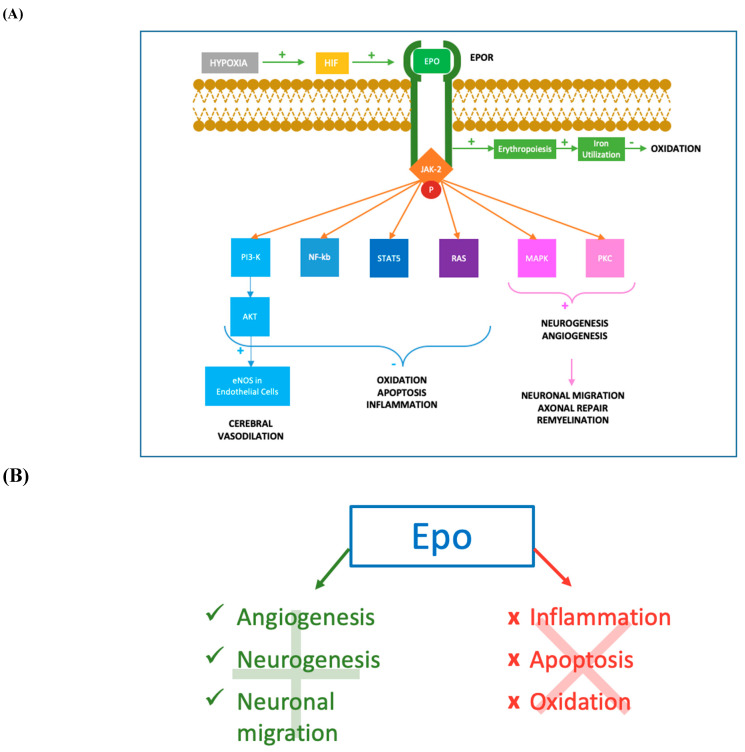
Molecular mechanisms of Epo action. (**A**) After hypoxia, the production of HIF determines an augmented synthesis of Epo which binds to its transmembrane receptor EpoR. The cytoplasmic tail of the Epo-EpoR complex phosphorylates JAK2, which activates a complex cascade of signalling pathways. The activation of PI3K/AKT pathway leads to the increase of eNOS activity in endothelial cells, determining augmented levels of NO and cerebral vasodilation. PI3K/AKT, together with NF-kb, STAT-5 and RAS pathways, reduces inflammation, oxidation and apoptosis acting both at nuclear and intramitochondrial levels. Moreover, MAPK and PKC mediated mechanisms promote neurogenesis and angiogenesis in damaged tissues. Neurogenesis, especially oligodendrogenesis, facilitates the process of remyelination and axonal repair, while angiogenesis permits flow restoration and migration of neuronal progenitors in the ischemic areas. The erythropoietic effect of Epo-EpoR binding is an additional antioxidant mechanism, increasing iron utilization resulting in a lower iron oxidant potential. JAK-2: Janus-tyrosine-kinase-2; PI3K: phosphoinositide 3-kinase; AKT: v-akt murine thymoma viral oncogene homolog; eNOS: endothelial nitric oxide synthase; NO: nitric oxide; NF-kb: nuclear factor kappa-light-chain-enhancer of activated B cells; STAT-5: signal transducer and activator of transcription 5; RAS: rat sarcoma; MAPK: mitogen-activated protein kinase; PKC: protein kinase C. (**B**) Mechanisms promoted and inhibited following Epo treatment. Epo has shown anti-apoptotic [73,74,75], antioxidant [76,77,78] and anti-inflammatory properties [79,80,81,82,83]. Epo promotes angiogenesis [74,86,87], oligodendrogenesis, neuronal progenitors migration in the ischemic areas (Wang et al., 2004; Ohab et al., 2006; Li et al., 2007) and neurogenesis [88,89,90,91].

Pleiotropic functions of Epo concerning neuroprotection have been studied in the last few decades. The first evidence for the extra-hematopoietic properties of Epo were published between 1993 and 2000 [67,68]. Normally, only a small percentage of circulating Epo is able to pass the BBB, binding the Epo receptor (EpoR) located on the capillary vessels [69]. However, it has been demonstrated that Epo is also produced in the developing brain, where it acts as a neuroprotective agent and growing factor [70,71,72]. In particular, Epo is primarily produced by astrocytes, followed by oligodendrocytes, endothelial cells, neurons and microglia, induced by hypoxia through the Hypoxia Inducible Transcription Factor pathway [70,71,72]. Figure 1. Both in animal models and in newborns, Epo has shown anti-apoptotic [73,74,75], antioxidant [76,77,78] and anti-inflammatory properties [79,80,81,82,83]. These actions can be exerted directly or mediated by a specific receptor named EpoR, which has been isolated in glial cells, neurons and brain endothelial cells in the hippocampus, cortex internal capsule and midbrain regions [70]. Molecular mechanisms following Epo treatment are shown in Figure 2. When not binding to its ligand, EpoR activates pathways leading to cell death. On the contrary, when Epo is bound to EpoR, cellular survival is promoted [84,85]. In addition, the role of Epo has also been described in promoting angiogenesis [74,86,87], oligodendrogenesis and neurogenesis [88,89,90,91]. In most of clinical studies, Epo is given in the form of recombinant human erythropoietin (rhEpo), which was the first purified and cloned [92]. Considering the difficulties in obtaining a sufficient quantity of Epo from the blood serum of living humans, rhEpo is produced in higher quantities from cultured mammalian hamster ovary cells and is highly purified [92,93]. At least nine isoforms of rhEpo can be identified, among which are epoetin alfa/alpha/beta, darbepoetin (Darbe) and carbamylated Epo [94]. Darbe is an analog of Epo with an additional sialic acid, able to confer a three-fold longer serum half-life compared with epoetin alfa, the most used isoform [95]. Epo and Darbe are termed as erythropoiesis-stimulating agents (ESAs) [96].

The aim of this review was to analyse the most recent evidence about the role of Epo as a neuroprotective strategy in the most common causes of neonatal neurological injury such as prematurity, HIE and perinatal stroke.

## 2. Materials and Methods

### 2.1. Research Strategy for Study Identification

A critical review was conducted using the 2020 PRISMA statement guidelines (www.prisma-statement.org, 15 December 2021), from February 2021 to May 2021, using the following databases: PubMed (2000–2021), Cochrane Trials Library CENTRAL (2000–2021). Additional ongoing studies were sought on the US National Institute of Health online registry for clinical trials (www.clinicaltrials.gov, 15 December 2021). The research strategy was conducted as follows: “erythropoietin” [All Fields] AND “neuroprotection” [All Fields] AND “newborns” [All Fields]. The results were combined with cross-referencing of previous reviews. 

### 2.2. Eligibility Criteria and Study Selection

Clinical trials exploring the potential role of Epo or Darbe for neuroprotection in newborns were selected. Study population included human preterms, full-term newborns suffering from HIE or affected by/at risk of neonatal stroke. 

After removing duplicates, studies were screened by title and abstract by three investigators independently. The following inclusion criteria were used: (1) human newborns as the study population, (2) use of Epo or Darbe as the only administered drug, (3) trials that reported effects of Epo or Darbe on neuroprotection as primary or secondary outcome.

Exclusion criteria were: (1) human adults or animal models as study population, (2) different outcomes from neuroprotection, (3) trials with Epo or Darbe given in association with other drugs, (4) not available full-text studies, (5) reviews, meta-analysis or journal editorials. The specific usage of Epo or Darbe was reported in each study represented on the Tables 1 and 2.

### 2.3. Data Extraction of the Studies

Data from the studies were collected using the “PICO” methodology (population, intervention, control and outcome).

## 3. Results

**Figure 3:**
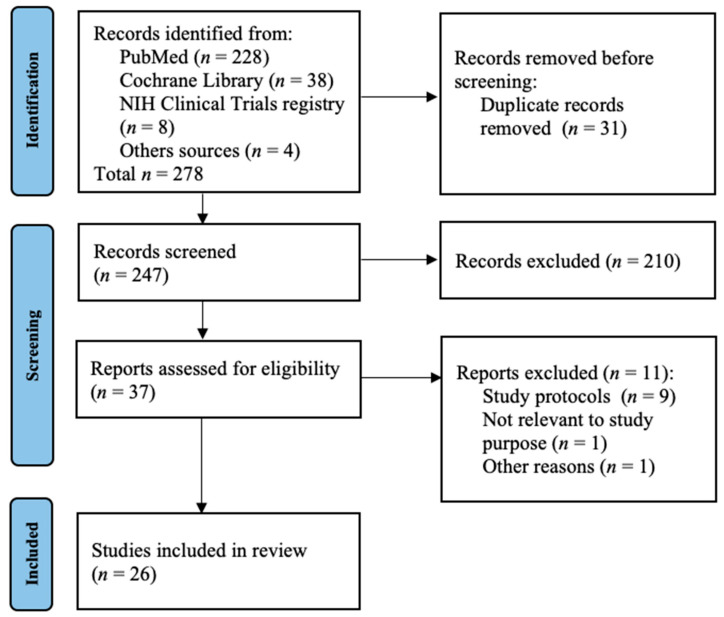
Study selection diagram.

A total of 278 records were identified. After adjusting for duplicates and inclusion criteria, a total of 37 trials were eligible for our study. Eleven studies were excluded after full-text assessment: nine studies were protocols for developing or ongoing studies without preliminary data; one study had outcomes which were irrelevant for the aim of the review. Finally, one paper was a pilot study whose findings were updated later in a trial already included in our review. Twenty-six trials were finally analysed: 16 trials about Epo effects in preterm population, 8 studies investigating the role of Epo in HIE and 2 studies concerning Epo administration in patients with stroke or at risk of it Figure 3.

### 3.1. Erythropoietin Administration in Preterm Infants

Sixteen studies investigated the neuroprotective role of Epo in preterm infants, for a total number of 2923 preterm babies with a gestational age at birth between 24–32 weeks or birth weight 500–1500 g. Two trials enrolled a group of term controls [98,99]. Table 1 reports target population, intervention, outcomes and findings.

Out of 16, seven studies described Epo intravenous (iv) administration in the first 2–3 days of life, with a dosage between 500–3000 UI/kg/dose [103,105,106,108,109,110,111]. Three studies evaluated the cumulative dose of Epo, 8574 U/kg [102] or 6300 UI/kg [107] or 3750 U/kg [101], given either iv or subcutaneous (sc) in the first 6-9 weeks of life. Four studies evaluated sc Epo at a dose of 400 UI/kg administered three times per week until 35 weeks of corrected postnatal age [98,99,100,104]. A single study evaluated a combination of Epo given iv and sc. Firstly the iv administration was given at a dose of 1000 UI/kg every 48 h for a total of six doses, subsequently sc doses of 400 UI/kg were provided for three times per week until 32 weeks of corrected postnatal age [112]. In eight trials Epo treatment was compared to placebo [100,103,105,106,108,109,111,113], while three protocols evaluated also the administration of Darbe, at a dose of 10 mcg/kg once a week until 35 weeks of corrected postnatal age [98,99,104].

A single study evaluated mortality rate and major adverse events as a primary outcome [108]. Safety of early high-dose of Epo administration was demonstrated, with no excess in mortality [108]. Furthermore, no differences in the incidence of IVH, ventricular dilatation, cystic and non-cystic PVL, sepsis, necrotizing enterocolitis, persistent ductus arteriosus ligation, bronchopulmonary dysplasia were reported [108]. In particular, no differences were reported in the incidence of ROP and infantile hemangioma in Epo treated babies compared to controls, as possible adverse effects of Epo administration [114,115]. An increased erythropoiesis was described as a result of the megakaryocytes shift towards the erythroid lineage, although hemocytometric values remained within the normal range [108].

Neurodevelopmental outcomes were evaluated in the first two years of life in seven studies [100,101,104,105,107,109,112]. Four studies considered a longer follow-up until 3–4 years [98,103], 5 years [113] or even 13 years [102]. In addition to standard neurological examination, anthropometric measures and the evaluation of vision and hearing abilities were analysed [98,100,101,102,103,104,105,107,109,112,113]. Neurodevelopmental outcomes were assessed through Hamburg-Wechsler intelligence tests for children—Third Edition (HAWIK-III—Tewes et al., 1999) score [102], Bayley scales of infant and development—Second Edition (BSID-II—Bayley, 1993) or Third Edition (BSID-III—Bayley, 2005) [100,101,103,104,109,112], Griffiths’ Mental Developmental Scales (Huntley, 1996) [107], Behavior Assessment System for Children—Second Edition (BASC-II—Reynolds et al., 2004) [98], Gross Motor Function Classification System (GMFCS—Palisano et al., 1997–2007) [112,113] and Mental Processing Composite of the Kaufman Assessment Battery for Children—First Edition (KABC—I, Rozenblatt, 2011) [113]. Five studies reported a beneficial effect of Epo treatment in at least one of the neurodevelopment scales used for the analysis. In Epo treated patients, an increased score at Motor Development Index (MDI) [100,101] or BSID [103,104] or HAWIK-III IQ score [102] was reported compared to controls. Two studies highlighted a dose-response correlation between Epo doses and higher MDI scores [100,101]. The longest-term—until 10–13 years—follow up study reported better anthropometric measures and sensorineural functions when newborns were treated with Epo [102]. In addition, significant cognitive improvements and OP scores (evaluation of Object Permanence, included in BSID III) were associated with Epo and Darbe administration [104]. Positive cognitive results were also reported in a subgroup of ELBW affected by IVH using the HAWIK-III IQ score [102]. On the other hand, four studies didn’t show any difference in neurodevelopmental outcomes in Epo treated patients compared to controls, assessed through Griffiths’ [107], MDI, Psychomotor Development Index (PDI) [109] and GMFCS [112] at 2 years of age, or GMFCS and KABC at 5 years of age [113]. 

Four studies performed brain MRI to evaluate the outcomes of brain injury. The MRI analyses highlighted a reduced structural brain damage in the Epo treated group [105]. In addition, an improved white matter development assessed was observed using DTI and TBSS [106] and a weak but widespread effect in the overall structural connectivity network after Epo treatment, measured on DTI [111]. Higher FA values at the posterior limb of the internal capsule, the splenium of the corpus callosum, frontal white matter, and occipital white matter were found in Epo treated group compared to controls [110]. 

On the other hand, the spectroscopy analysis showed no difference for any brain metabolite level neither in premature nor in ESAs treated babies [99]. There was no significant difference in FA values at the parietal white matter, thalamus, lenticular nucleus, and caudate nucleus between Epo treated and control group [110].

### 3.2. Erythropoieti Administration in HIE

Eight studies investigated the neuroprotective role of Epo in newborns with HIE, for a total number of 533 patients. In most of these studies, patients enrolled had a gestational age ≥ 37 weeks [116,117,118,119,120], one study considered patients ≥ 40 weeks [121] and two studies ≥ 36 weeks [122,123]. All newborns were affected by HIE according to Sarnat score [124]. Table 2 reports target population, intervention and outcome.

Erythropoietin was administered in the first weeks of life, at different doses between 200–2500 U/kg/dose. Concerning the administration scheme, in three studies Epo was given every two days for a total of 12, 7, 6 or 5 doses [116,118,119,120]. In three studies Epo was given once a day for the first three days of life [117,122,123]. Two more doses at day 5 and 7 were administered in two of these protocols [122,123]. In one study Epo was given as a single administration [121]. 

All the studies evaluated Epo safety, reporting mortality rate and/or adverse events and/or the incidence of disability. None of them reported an increase in mortality or disability rate in patients treated with Epo. Erythropoietin was well tolerated, without allergic reactions, venous thrombosis or alterations of electrolytes, liver and renal functions [116]. Erythropoietin was not related to higher incidence of hypotension, hepatic dysfunction, prolonged coagulation, necrotizing enterocolitis, hypertension, polycythemia or thrombosis [119]. As a consequence of Epo hematopoietic effect, patients treated with Epo had higher levels of haemoglobin and erythrocytes compared to controls [116].

In two studies Epo therapy was associated with TH in comparison with TH alone [122,123]. The effect of different doses of Epo administered with TH was also described [118]. Only one study enrolled healthy controls [121]. Study protocols comparing Epo with placebo or only supportive therapy, reported a lower death rate [117,119,121] and disability index, evaluated with GMFCS, visual and hearing assessment [119] or BSID at 18 months [117]. Moreover, Epo alone decreased brain injury at MRI analysis performed at 10–14 days of life [119].

Compared to TH alone, The Epo effect was controversial. In fact, in one study Epo treatment compared to TH alone in moderate HIE showed a lower mortality and disability rate, together with higher scores at 18 months BSID-II [116].

Yet, another study described a weaker effect of Epo treatment on mortality rate and disability compared to TH alone [121]. The combined treatment of Epo and TH was found to be safe, not worsening brain damage at 4–13 days MRI nor affecting disability evaluated with GMFCS and neurodevelopment assessed through BSID-II or III at 22 months [118]. Erythropoietin associated with TH determined a lower mortality rate [122], a lower brain injury score at MRI performed between 4–7 days of age [122,123] and a better neurodevelopmental outcome with higher score of AIMS and WIDEA at 12 months when compared to TH alone [122]. Furthermore, the combined Epo and TH group improved neurodevelopmental outcomes assessed with Warner Initial Developmental Evaluation (WIDEA-Msall, 2006) and Alberta Infant Motor Scale (AIMS, Piper, 1994) at 12 months and had a smaller volume of acute brain injury assessed with MRI at ≤7 days of life [123]. 

Erythropoietin treatment in neonates with moderate and severe HIE showed better physical and mental developmental indexes at 6 months of age compared to controls [120].

No differences in terms of mortality in Epo treatment compared to phenobarbital administration were found and both therapies determined a reduced death rate compared to controls [117]. The antioxidant effects of the treatments were evaluated through the dosage of antioxidant enzymes Superoxide Dismutase and Glutathione Peroxidase, Total Serum Antioxidant Status and Malondialdehyde, as markers of free radical damage [117]. Plasma levels of Superoxide Dismutase and Glutathione Peroxidase were lower in Epo and Phenobarbital groups, while Total Serum Antioxidant Status levels were higher, especially in Epo compared with Phenobarbital group. However, Malondialdehyde did not show any significant difference in the groups [117]. A lower mortality rate in Epo or phenobarbital treatment was reported and a better cognitive and motor function assessed through BSID-II at 18 months of age was also described [117]. 

A single study evaluated the effect of TH or Epo or supportive therapy on serum brain-derived neurotrophic factor and neuron-specific enolase concentration, compared to healthy controls [121], reporting an increased brain-derived neurotrophic factor and decreased neuron-specific enolase level in treated groups on day 5 compared to day 1 [121].

### 3.3. Erythropoietin Administration in Neonatal Stroke

Two studies investigated the effects of Epo administration in children with neonatal stroke [125] or at risk of stroke in the neonatal period in a population of neonates scheduled for cardiac surgery with hypothermic cardiopulmonary bypass [126]. A total of 80 full-term newborns were enrolled. Table 3 reports target population, intervention and outcome.

Erythropoietin was administered intravenously in both trials, at a dose of 1000 UI/kg, except for twenty-six patients who received 500 UI/kg [126]. In Benders’ study Epo was administered for three days straight after radiological diagnosis of idiopathic perinatal arterial ischemic stroke [125]. In Andropoulos’ study, Epo was given in three doses at 1000 UI/kg or 500 UI/kg pre and post-surgery [126].

In both trials, the safety profile of Epo administration was registered and effects of Epo were evaluated with brain MRI exams and neurodevelopmental assessment. None of them highlighted a significant benefit on neuroprotection in Epo treated children vs. placebo groups [125,126]. 

Both studies analysed the safety profile of EPO administration and no significant differences in adverse events or death rates were detected in Epo or placebo groups. In particular, in both studies vital signs and biochemistry blood parameters remained within the normal range. Andropoulos registered six cases of postoperative dural sinovenous thrombosis, equally distributed in Epo and control group [126]. 

In Benders et al. study, brain MRI was performed after clinical and ultrasound suspicion of perinatal arterial ischemic stroke to confirm diagnosis and then repeated at 3 months of age [125]. In the other study, MRI imaging was executed as preoperative assessment and then as a control 7–10 day after cardiac surgery [126]. The brain MRI evaluation of both studies showed comparable results of neuroimaging performed at 7–10 days [126] and 3 months [125] in the Epo treated group and the controls. In particular, no difference on intracranial haemorrhage, thrombosis, infarction or white matter injury occurrence was described in Epo treated population [126]. Even in Benders’ follow-up study, any significant discrepancy was highlighted at the age of 3 months in the percentage of the stroke tissue that had dissolved [125] and no difference in the total brain volume was found in Epo treated and controls [125].

Neurodevelopmental outcome was assessed, respectively at 3 months follow up with Griffiths’ scale [125] and at one year follow up with BSID-III [126]. None of the two trials found any statistically difference between Epo treated and placebo groups.

## 4. Discussion

### 4.1. Erythropoieti in Preterm Infants

Erythropoietin has been administered to preterm infants since 1990 for haematological purposes. In fact, Epo has been extensively used for the prevention of anemia of prematurity, but the discovery of the potential role as a neuroprotective agent suggested new purposes for its administration [127,128,129]. 

In the last few decades the survival of preterm infants has dramatically grown, yet this population is still exposed to a great incidence of neurodevelopmental impairment [7,8]. Trials conducted in animal models have shown neuroprotective effects of Epo, thus clinical studies investigating Epo’s new potential benefits in premature babies have followed [130,131,132,133,134]. In particular, it has been described that Epo administration in preterm is able to protect neurons and oligodendrocytes from apoptosis, prevent inflammation and promote neurogenesis and angiogenesis [135,136]. Preterm infants benefit by a prolonged treatment during the period of oligodendrocyte maturation, between 24 and 32 weeks [2,130,137], in which Epo exerts a neuroprotective function. Evidence in newborns and animal models suggest neuroprotective effects at doses >200 U/kg [138]. Cumulative doses have been associated with more beneficial effects compared to a single-dose administration [139]. 

The dosage of EPO ranged from 250 to 3000 UI/kg in all studies. Darbe alfa was administered at 10 mcg/Kg sc [98,99,104].

The population target was represented of extremely and very preterm babies (gestational age below 32 weeks or BW < 1500 gr). Only three clinical trials focused on the role of Epo in extremely preterm infants (gestational age below 28 weeks or 1000 gr) [102,103,112].

Erythropoietin administration was proved to be safe in all preterm infants, not affecting mortality rate and major adverse effects occurrence in the short and long term [103,108,112]. These data confirmed previous analyses on animal models [139,140,141].

Out of 16, 10 trials performed clinical neurodevelopmental outcome evaluated mainly in the first years of life. No uniform results were reported. 

In addition, neurodevelopmental outcomes were not assessed in all trials in which neuroimaging was performed. It has been well recognized that a correlation between white matter injuries and neurodevelopmental outcome, in terms of increased incidence of cerebral palsy, motor and cognitive delay exists [15,16,142,143]. In addition, values of fractional anisotropy of the corpus callosum assessed using TBSS have been reported to directly correlate with motor and cognitive outcomes [144]. Thus, neuroimaging alterations have been related with poorer neurodevelopmental outcomes [95,98,99,106,145]. Studies on Epo treated very preterm newborns highlighted an improved white matter development and a weak but widespread effect in the overall structural connectivity network after Epo treatment [110,111]. Yet, no difference for any brain metabolite has been reported in very preterm treated group [99]. 

### 4.2. Erythropoietin in HIE

After several years of studies conducted in animal models, in 2009 the first trial of Epo administration as adjuvant therapy with TH in newborns affected by HIE was published. The study underlined Epo favourable effects, paving the way to further studies [116]. Since then Epo showed beneficial effects during the secondary energy failure and the tertiary phases of HIE [146]. 

The therapeutic scheme is influenced by the type of damage [2]. In HIE term newborns high Epo doses are more effective when the injury has not been established yet [2,130,137]. Evidence from animal models have shown that Epo should be administered at high doses within 6 h after the onset of brain injury to reach a substantial neuroprotective effect [147]. In this context, Epo may influence the mechanisms of cerebral flow restoration, neovascularization and neuroregeneration, limiting the ischemic damage [148]. 

Our analysis revealed that Epo administration was safe and well tolerated [116], confirming previous findings in animal models [132,139,141]. 

The passage through the BBB is an essential issue to be considered for any neuroprotective drug given systemically. Erythropoietin presents a molecular weight too high to be carried across the BBB through the lipid mediated transport [116]. Transport of Epo through the BBB is mediated by a specific receptor-facilitated process [69] as well as a time, dose and peak serum concentration-dependent mechanism [149,150]. Furthermore, the alteration of BBB permeability caused by asphyxia determines in patient with HIE an easier passage from serum to cerebrospinal fluid [151]. In the studies analysed, Epo administration is followed by a higher concentration of Epo in the cerebrospinal fluid compared to HIE controls, thus confirming the increased levels are due to the contribution of Epo exogenous administration [116].

Administration of Epo has shown some advantages compared to TH treatment. Treatment with Epo implies easier technical performance, with less side effects [116,121]. Moreover, Epo treatment is effective if administered within 48 h of life, differently from TH which must be initiated within the first 6 h of life [116]. Thus, Epo treatment has been proposed as both an alternative therapy or combined treatment with TH [116,121].

In all the protocols comparing Epo vs. placebo or Epo associated with TH vs. supportive therapy, Epo treatment determined a lower death rate and improved neurologic outcomes [117,119,120,121,122,123]. Valera et al. treated 15 HIE neonates with EPO for two weeks, starting within three hours from birth along with TH. At 18 months, there was 80% survival with no neurodevelopmental disability. Unfortunately, there was no control group for comparison [152]. 

Rogers et al. recruited 24 newborns with HIE and administered Epo 24 h after HIE combined with standard TH. The authors found that significant neurodevelopmental disability occurred in only 12.5% of infants with moderate to severe MRI changes who received Epo plus TH, compared to 70–80% significant disability or death in infants treated with TH alone. However, these results were not statistically significant as the number of infants was too small [118]. The Phase II clinical trial on newborns with HIE reported better 12-months motor outcomes for treatment with Epo plus TH compared to TH alone [123]. A randomized case-control study by Wu et al. [122] evaluating 24 newborns treated with Epo and TH for moderate/severe HIE, showed a significantly reduced brain injury on MRI at 5 days-of-age and better 12-months motor outcomes compared to the 26 infants who received TH as a standard routine measure for HIE. The Epo treatment was also associated with an antioxidant effect and an increase of neuroprotective factors [117,121]. The study by Wang et al. clearly demonstrated the protection from the damage resulting from asphyxia through inhibiting apoptosis, reducing oxygen free radicals and enhancing antioxidant capacity. In this research trial, 34 newborns received Epo and Vitamin C plus TH compared to 34 newborns receiving conventional treatment plus ascorbic acid. There were lower oxidative stress index levels and higher antioxidant enzymes in both groups after treatment, with better results in the group treated with Epo. Moreover, lower pro-apoptotic molecules and higher anti-apoptotic molecules were found in both groups after treatment, with better results again in the group treated with Epo [153].

In addition, MRI analysis confirmed the beneficial effect of Epo in the reduction of brain injury, both if administered alone [119] or combined with TH [123]. Despite all these data, the optimal dosage and administration regimens for the treatment of HIE in newborns still needs to be defined [116,118,121].

### 4.3. Erythropoietin in Neonatal Stroke

At the diagnosis of neonatal stroke, the damage has already been established and the reparation mechanisms are not fully known, thus nowadays acute therapies for perinatal strokes are still not available [154]. Currently the management of the disease is mainly supportive, based on the treatment of complications, such as antiepileptic therapy, and on the improvement of neurologic chronic sequelae through rehabilitation [155]. Erythropoietin has become one of the most remarkable neuroprotective strategy since Sakanaka et al. reported for the first time the role of Epo as a neuroprotective agent in the ischemic brain damage [156]. Recent researches are focused on new approaches including the administration of adjuvant neuroprotective therapies, with the aim to enhance endogenous mechanisms of repair and angiogenesis [157,158]. 

In our review, the safety profile of Epo administration was also confirmed in the studies on neonatal stroke. This is consistent with previous data available from animal models and preterm and HIE-suffering newborns, which showed a good tolerance of treatment with Epo in those populations [128,147,159,160]. 

The brain MRI evaluation in both studies didn’t find a beneficial effect of Epo treatment on stroke volume [125,126]. Furthermore, Epo treatment was not associated with an overall difference in neurodevelopmental outcome, at 3 months and one year follow up respectively with Griffiths’ scale and BSID-III [125,126]. 

These data are in contrast with most of the newborn stroke animal models in which several neuroprotective effects and reduction of stroke volume induced by Epo are described [61,130,161,162]. Recently, a delayed Epo therapy administered to mice model with middle brain artery occlusion stroke reported to uphold brain volume and to improve behavioural and sensorimotor functions, compared with placebo-treated animals [61]. 

The discrepancy between the experimental animal model and clinical trials may be due to the small neonatal study population, as the trials are both phase I and II studies. Furthermore, Andropoulos and colleagues found that six enrolled newborns presented microdeletions at the chromosome 22q11.2 region, a condition known to be linked to a variable degree on neurodevelopmental impairment in a syndromic condition [163]. 

Another limiting factor is the therapeutic window, as the beneficial effect is limited to specific time after injury [164]. One interventional study used Epo after stroke diagnosis, which is usually done 24–48 h after cerebral vascular accident. The use of specific biomarkers that will increase within the first hours of life in hypoxic-ischemic injury may help in the early diagnosis of stroke, allowing a prompt identification of neonates who may qualify for neuroprotection.

Finally, the variability in period and tests used to assess the neurodevelopmental outcome may represent a weakness and suggests that scores should be interpreted with caution. Since the determinants of cognitive outcomes at school age are multifactorial, the predictive value of tests administered at age < 24 months might never approach 100%.

## 5. Conclusions

Brain injury still represents an open challenge in the neonatal setting. Therapeutic hypothermia has become the standard therapy in term newborns suffering from perinatal asphyxia. Since 2009, Erythropoietin (Epo) has been identified as a pharmacological compound playing an important role in preserving the developing brain. Clinical trials reveal that Epo can be employed as one of the therapeutic treatments of perinatal asphyxia either as an adjuvant therapy with hypothermia or a substitute for hypothermia in HIE. No beneficial effect in improving morbidity was observed after Epo administration in perinatal stroke.

When administered in preterm infants, some clinical studies report beneficial effect on neurodevelopmental outcome, others do not show efficacy.

An early diagnosis and a shorter latency time to treatment are essential for successful outcomes both in preterm and term newborns suffering from hypoxic-ischemic injury. Further studies focused on tailored regimens accounting for risk stratification of brain damage are needed. Moreover, the identification of specific markers and their temporal expression at different time of recovery after hypoxic-ischemia may be implemented to optimize the Epo therapeutic scheme in the developing brain. 

## Figures and Tables

**Table 1:** Erythropoietin (Epo) administration in preterm infants.

First Author (Year)	Target Population	Intervention	Outcomes	Findings	Ref.
Bierer (2006)	GA: ≤32 weeks BW 401–1000 g N = 12	Epo 400 U/kg 3 times/week, from the 4th day of life to 35th postmenstrual week (n = 6) (n = 6 placebo)	Evaluation at 18–22 months Anthropometric measurements BSID II Neurologic examination Incidence of neurodevelopmental impairment	No difference No difference in PDI, higher MDI score in patients with higher Epo concentrations No difference No difference	[100]
Brown (2009)	GA: ≤30 weeks BW < 1500 g N = 82	Epo 3 times/weeks for 6 weeks, mean cumulative dose 3750 U/kg (range 250–400 U/kg/dose) firstly iv then sc.	Evaluation at 22 months PDI and MDI scores of BSID II.	Relationship between Epo doses and PDI and MDI scores of BSID II.	[101]
Neubauer (2010)	BW ≤ 1000 g N = 146	Epo at a cumulative 8574 U/kg administered over 68 days sc or iv (n = 89) (n = 57 no treatment)	Evaluation at 10–13 years Neurodevelopmental/sensorineural outcome Cognitive outcome defined by IQ, determined with HAWIK-III; Anthropometric measurements.	Better neurodevelopmental outcome in Epo treated group, lower incidence of major impairment. No difference in sensorineural outcome. Higher IQ scores at 10–13 years in Epo treated patients. Better values of head circumference in Epo treated group, no effect on weight and height.	[102]
McAdams (2013)	BW ≤ 1000 g N = 35	Epo 500 U/kg, 1000 U/kg or 2500 U/kg once a day for the first 3 days of life (n = 17) (n = 18 no treatment)	Evaluation at 4–36 months Anthropometric outcomes Neurodevelopmental outcome through BSID II or III Hearing and vision status.	No difference Modest improvement in cognitive and motor scores in Epo group No difference	[103]
Ohls (2014)	BW 500–1250 g N = 80	Darbe alfa 10 mcg/kg once a week sc (n = 27); or Epo 400 U/kg, 3 times a week sc, until 35 weeks of postnatal corrected age (n = 29) (n = 24 placebo)	Evaluation at 18–22 months: Language and social-emotional scores alone Evaluation of OP and early working memory at BSID II.	Epo and Darbe treated groups had higher cognitive scores compared to placebo group at BSID II. Treated groups had higher OP scores, with a positive trend in language skills. Darbe group better OP scores than Epo group. No differences were found for other outcomes.	[104]
Leuchter (2014)	GA 26–31 weeks N = 165	Epo 3000 IU/kg at <3 h, 12–18 h and 36–42 h after birth (n = 77) (n = 88 placebo)	Evaluation at term equivalent age MRI alterations	Reduced structural brain damage at MRI acquired at term-equivalent age in Epo treated group	[105]
O’Gorman (2015)	GA 26–31 weeks N = 58	Epo 3000 IU/kg at <3 h, 12–18 h and 36–42 h after birth (n = 24) (n = 34 placebo)	Evaluation at term equivalent age MRI alterations (white matter development, using DTI and TBSS)	improved white matter development in Epo treated group assessed through DTI and TBSS.	[106]
Luciano (2015)	GA ≤ 30 weeks N = 104	Epo at a median cumulative dose of 6300 UI/Kg (6337 ± 2434 UI/Kg) for 6.9 ± 2.4 weeks, starting at age of 4 days (n = 59) (n = 49 not treated)	Evaluation at 24 months neurodevelopmental quotient through Griffiths’ Mental Developmental Scales	No improvement in neurodevelopmental outcome	[107]
Fauchère (2015)	GA 26–31 weeks N = 443	Epo 3000 IU/kg at <3 h, 12–18 h and 36–42 h after birth (n = 229) (n = 114 placebo)	Evaluation between 7–10 days of age Mortality rate Incidence of major adverse neonatal complications	No difference No difference	[108]
Natalucci (2016)	GA 26–31 weeks N = 450	Epo 3000 IU/kg at <3 h, 12–18 h and 36–42 h after birth (n = 225) (n = 225 placebo)	Evaluation at 2 years of corrected age anthropometric parameters MDI, PDI score, incidence of cerebral palsy, severe hearing and visual impairment	No difference No difference	[109]
Lowe (2017)	BW 500–1250 g N = 71	Darbe alfa 10 mcg/kg once a week sc or Epo 400 U/kg, 3 times a week sc, or placebo until 35 weeks of postnatal corrected age (n = 35) (n = 14 placebo preterm; n = 22 full term controls)	Evaluation at 3.5–4 years behavioural outcomes at the four components of BASC-2: adaptive skills, behaviour symptoms, externalizing problems, internalizing problems	Beneficial role of EPO and Darbe on behavior in preterm infants with low socioeconomic status. The finding was independent from the effect on cognition	[98]
Gasparovic (2018)	BW 500–1250 g N = 52	Darbe alfa 10 mcg/kg once a week sc or Epo 400 U/kg, 3 times a week sc, until 35 weeks of postnatal corrected age (n = 15) (n = 15 placebo preterm) (n = 22 term controls)	Evaluation 4 and 6 years of age 1H-MRS spectra from the anterior cingulate (grey matter) and frontal lobe white matter, assessing combined N-acetylaspartate and N-acetylaspartylglutamate (tNAA), myo-inositol, choline compounds (Cho), combined creatine and phosphocreatine, and combined glutamate and glutamine	No significant differences for any investigated metabolite level; Significant age-related increases in white-matter tNAA and Cho, increased grey-matter tNAA.	[99]
Yang (2018)	GA < 32 weeks, BW < 1500 g N = 81	Epo 500 U/kg dose within 72 h from birth every 48 h for 2 weeks (n = 42) (n = 39 term controls)	Evaluation at term equivalent age: MRI alterations (white matter development, using fractional anisotopy (FA) of DTI.	Higher FA values at the posterior limb of the internal capsule, the splenium of the corpus callosum, frontal white matter, and occipital white matter in treated group. No significant difference in FA values at the parietal white matter, thalamus, lenticular nucleus, and caudate nucleus between the two groups.	[110]
Jakab (2019)	GA 26–31 weeks N = 58	Epo 3000 IU/kg at <3 h, 12–18 h and 36–42 h after birth (n = 24) (n = 34 placebo)	Evaluation at term equivalent age: MRI alterations (overall structural brain connectivity, using DTI)	Weak effect of Epo treatment on the overall structural brain connectivity. Increase of local structural connectivity strengths in Epo treated infants, not limited to lobes of the brain.	[111]
Juul (2020)	GA 24^+0^–27^+6^ weeks N = 741	Epo iv 1000 U/kg every 48 h for a total of six doses, followed by sc maintenance of 400 U/kg 3/week up to 32 weeks of postmenstrual age (n = 376) (n = 365 placebo)	Evaluation at 22–26 months death or severe neurodevelopmental impairment; death or moderate-to-severe neurodevelopmental impairment; incidence of adverse events and common complications of prematurity	No difference No difference No difference	[112]
Natalucci (2020)	GA 26–31 weeks N = 345	Epo 3000 IU/kg at <3 h, 12–18 h and 36–42 h after birth (n = 177) (n = 168 placebo)	Evaluation at at 5 years of age Neurodevelopmental outcome assessed through the evaluation of general intelligence, using the KABC somatic growth, occurrence of cerebral palsy through GMFCS and severe hearing and visual problems	No difference No difference	[113]

**Table 2:** Erythropoieti (Epo) administration in HIE.

First Author (Year)	Target Population	Intervention	Outcomes	Findings	Ref.
Zhu (2009)	GA > 37 weeks BW > 2500 g moderate/severe HIE N = 153	Epo at 300 U/kg every two days for 2 weeks (n = 45) or 500 U/kg every two days for 2 weeks (n = 28) Hypotermia (n = 84)	Evaluation at 18 months Epo administration impact on mortality BSID-II	Minor rates of death Minor rate of disabilities and delayed IQ in newborns with moderate HIE	[116]
Wang (2011)	GA > 37 weeks moderate or severe HIE N = 70	Epo 200 U/kg/dose 3 times weekly for 2–4 weeks n = 35 (n = 22 moderate and n = 13 severe) Controls n = 35 (n = 24 moderate and n = 11 severe)	Evaluation at 28 days and 3, 6 months: Neonatal Behavioural Neurological Assessment (NBNA) at age of 28 days. The infant development test of Child Development Centre of China (including PDI and MDI) at ages of 3 months and 6 months	Better neonatal behavioural neurological assessment at 28 days of age in Epo group Higher PDI and MDI scores at 3 and 6 months	[120]
Avasiloaiei (2013)	GA ≥ 37 weeks; perinatal asphyxia N = 67	Epo 1000 U/kg per days for the first three days after birth (n = 22) Phenobarbital a 40 mg/kg single dose (n = 22) Supportive therapy (n = 23) No hypothermia was available	Evaluation at 3-6-9-12 months mortality rate neurodevelopment outcome with BSID-II.	Lower mortality rate Better cognitive and motor function in phenobarbital group at 3 and 6 months of follow up, no statistical difference at 12 months	[117]
El Shimi (2014)	GA ≥ 40 weeks HIEN = 45	Epo 1500 U/kg at day 1 of life (n = 10) Moderate hypothermia (n = 10) Supportive therapy (n = 10) (n = 15 controls)	Evaluation at 3 months: safety and efficacy of single dose mortality rates, MRI findings and neuromuscular function	No major side effects in newborns treated with Epo. Hypotermia group had less mortality rate than Epo group and supportive care Tendency to better scores in MRI scores and neuromuscular function scales at 3 months of follow up in hypothermia group compared to single-dose Epo group	[121]
Rogers (2014)	GA ≥ 37 weeks HIE N = 24	Epo 250 U/kg (n = 3) or 500 U/kg (n = 6) or 1000 (n = 7) or 2500 U/kg (n = 8) every 48 h for 6 times, starting from day 1 of life	Evaluation at 22 months BSID-II/III	No worsening on neurodevelopmental outcomes in Epo + hypothermia treated patients with HIE	[118]
Wu (2016)	GA ≥ 36 weeks; moderate or severe HIEN = 50	Hypothermia and placebo (n = 26) at day 1, 2, 3, 5 and 7 of life hypothermia and Epo (n = 24) 1000 U/kg at day 1, 2, 3, 5 and 7 of life	Evaluation at 12 months behavioural and neurodevelopmental outcome with WIDEA and AIMS scales at 12 months neonatal death rates brain injury at 3 and 7 days MRI	EPO group high scores at WIDEA and AIMS and assessments No differences on neonatal death rates EPO group minor signs of brain injury at MRI	[122]
Malla (2017)	GA ≥ 37 weeks; moderate or severe HIE	Epo 500 IU/kg on alternate days for a total of five doses with first dose < 6 h of age (n = 50) 2 mL of normal saline solution on alternate days for a total of five doses with first dose < 6 h of age (n = 50) No hypothermia was given	Evaluation at 10–14 days and 19 months death rates moderate/severe disabilities (including cerebral palsy, cortical visual and hearing impairments) brain injury at 10th to 14th day MRI	Epo group less rates of death, Epo group: less rates of death moderate/severe disabilities, cerebral palsy and need of anticonvulsivants treatment at mean 19 months of assessment. Epo group: less brains abnormalities at MRI	[119]
Mulkey (2017)	GA ≥ 36 weeks; moderate or severe HIEN = 24	Hypothermia and placebo (n = 24) at day 1, 2, 3, 5 and 7 of life Hypothermia and Epo (n = 20) 1000 U/kg at day 1, 2, 3, 5 and 7 of life (n = 11 received 3 doses, n = 8 received 4 doses, n = 1 received 5 doses before MRI assessment)	O: evaluation at ≤7 days and 12 months brain injury at ≤7 days MRI neurodevelopment outcome with WIDEA and AIMS	Epo group: less brain damage volume at MRI Higher brain damage volume in placebo group correlated with worse neurodevelopmental outcome	[123]

**Table 3:** Results of Erythropoietin (Epo) administration in neonatal stroke.

First Author (Year)	Target Population	Intervention	Outcomes	Findings	Ref.
Andropoulos (2013)	GA > 37 weeks, diagnosis of cardiac abnormalities scheduled for hypothermic cardiopulmonary bypass (CPB) for more than 60 minutes N = 42	Epo at 1000 U/kg over 60 min 12–24 h preoperatively; immediately after CPB and 24 h after dose 2 Or Epo 500 IU/ kg preoperatively and at days 1 and 3 post-surgery every two days for 2 weeks (n = 20 placebo)	Evaluation pre and post surgery and 22 months: Safety of Epo administration Evaluation of MRI brain injury pre- and post-surgery Neurodevelopmental outcome with BSID-III.	No major side effects in treated newborns No difference in pre and post-surgery brain MRI findings No difference in neurodevelopmental outcome	[126]
Benders (2014)	Neonates with a MRI confirmed perinatal arterial ischemic stroke N = 20	Epo 1000 U/kg immediately after MRI diagnosis and at 24 and 48 h after the first dose (n = 10) (n = 10 full term controls)	Evaluation at 3, 12 and 24 months Safety of Epo administration brain injury findings at three months MRI follow up neurodevelopmental outcome with Griffith’s scale at 12–24 months	No major side effects in treated newborns No beneficial effect of Epo on stroke volume at 3 months MRI No difference in motor and cognitive development assessments.	[125]

## Abbreviations

1H-MRS	Proton Magnetic Resonance Spectroscopy
AIMS	Alberta Infant Motor Scale for motor functions
BASC2	Behavior Assessment System for Children—2nd edition
BBB	blood brain barrier
BSID-II or III	Bayley Scales of Infant Development-II or III edition, composed by three scores—MDI, PDI and Infant Behavior Record
BW	birth weight
Darbe	darbepoetin
DTI	Diffusion Tensor Imaging
ELBW	Extremely Low Birth Weight
Epo	Erythropoietin
EpoR	Erythropoietin receptors
GMFCS	Gross Motor Function Classification System
HAWIK-III	Hamburg-Wechsler Intelligence Test for Children-III edition
HIE	Hypoxic Ischemic Encephalopathy
IQ	Intelligence Quotient
Iv	intravenous
IVH	Intraventricular Haemorrhage
KABC	Kaufman Assessment Battery for Children
MDI	Motor Development Index
MRI	Magnetic Resonance Imaging
NMS	Neuromuscular Function Scale
OP	Object of Permanence
PDI	Psychomotor Development Index
Sc	subcutaneous
TBSS	Tract Based Spatial Statistics
TH	Therapeutic Hypothermia
WIDEA	Warner Initial Developmental Evaluation
